# Agro-Industrial Waste from *Pistacia vera*: Chemical Profile and Bioactive Properties

**DOI:** 10.3390/plants14101420

**Published:** 2025-05-09

**Authors:** Mauricio Piñeiro, Victoria Parera, Javier E. Ortiz, Olimpia Llalla-Cordova, Sofia Manrique, Brisa Castro, Maximiliano Ighani, Lorena C. Luna, Gabriela E. Feresin

**Affiliations:** 1 Instituto de Biotecnología, Facultad de Ingeniería, Universidad Nacional de San Juan (UNSJ), San Juan 5400, Argentina; mauridpg@gmail.com (M.P.); victoriaparera.t@gmail.com (V.P.); sofiamanrique.2@gmail.com (S.M.); brisacastro3445@gmail.com (B.C.); lorenaluna@unsj-cuim.edu.ar (L.C.L.); 2 Consejo Nacional de Investigaciones Científicas y Técnicas (CONICET), CCT CONICET, San Juan 5400, Argentina; olimpiallcr@gmail.com; 3 Pisté S.R.L., San Juan 5400, Argentina; maximilianoighani@gmail.com

**Keywords:** nematicidal, *Meloidogyne incognita*, phytotoxicity, antioxidant, anticholinesterases

## Abstract

In Argentina and globally, pistachio (*Pistacia vera*) production has significantly grown, driven by its high nutritional value and food industry demand. Its harvesting and processing generate about 40% of pistachio waste (PW), including leaves, twigs, seed coats, green, and empty kernels. Underutilized PW has led to environmental problems, including soil and water contamination by landfill accumulation. However, it could be a potential source of undiscovered bioactive compounds. This study aimed to characterize the chemical profile and to evaluate the bioactive properties of PW. The dried pistachio waste (dPW) was used to prepare the pistachio waste decoction (PWD) (10% *w*/*v*). The total phenolic content (TPC) and flavonoid content (FC) were quantified, and the chemical profile was analyzed using UPLC-DAD-ESI-MS/MS. Nematicidal activity against *Meloidogyne incognita* (J2), acetylcholinesterase (AChE) and butyrylcholinesterase (BuChE) inhibition, antioxidant capacity (ABTS, DPPH, FRAP), and phytotoxicity on *Allium cepa*, *Lactuca sativa*, and *Raphanus sativus* were evaluated. The UPLC-DAD-ESI-MS/MS analysis identified 26 compounds, including phenolics, flavonoids, and unsaturated fatty acids. The main compounds were gallic acid, anacardic acid, and quercetin derivatives. The TPC and FC were 212.65 mg GAE/g dPW and 0.022 mg QE/g dPW, respectively, displaying strong antioxidant activity across the assays DPPH, ABTS, and FRAP. PWD exhibited nematicidal activity against *M. incognita* (J2) (LC_50_ = 0.12% at 24 h). Alterations in the cuticle were observed, including structural disorganization and detachment from internal tissues. Additionally, a remarkable cholinesterase inhibitory effect was detected at 2.0% PWD (42.65% for AChE and 58.90% for BuChE). PWD showed low phytotoxic effects across the tested species, and the germination percentage (GP) and the mean germination time (MGT) were not significantly affected (GP > 79%). These findings highlight the potential of PW as a sustainable alternative for *M. incognita* control, the remarkable nematicidal, anticholinesterase, and antioxidant properties, and the low phytotoxicity, supporting its use in sustainable agricultural practices.

## 1. Introduction

The pistachio (*Pistacia vera* L.), a member of the Anacardiaceae family, produces a nut with a high value, widely consumed for its exceptional nutritional profile and unique sensory attributes [1]. Over the past decades, global pistachio production has expanded considerably, exceeding 1300 kilotons in 2023 [2]. However, large-scale production generates substantial amounts of pistachio waste (PW), including seed coats, soft hulls, and hard shells [3]. Throughout the harvesting process, the trees are mechanically shaken, and in the subsequent fruit separation stage, the hulls are removed using an abrasive peeler. Significant amounts of waste are generated, including remnants of leaves, twigs, and non-commercial fruits (green and empty kernels). These underutilized residues lead to multiple environmental challenges, including soil and water contamination, air pollution, and the release of greenhouse gases due to landfill accumulation.

Phiri et al. [4] highlights innovative strategies for sustainable agro-waste management, advocating for a shift from the linear “take–make–dispose” model to the reuse, recycling, and resource recovery promoting systems. The increasing demand for sustainable practices has fueled the interest in the valorization of agro-industrial by-products, particularly as a source of bioactive compounds. Maccarronello et al. [5] emphasized that agricultural by-products are rich in phytochemicals with potential applications in the food, pharmaceutical, and agricultural industries. PW is a promising source of bioactive molecules, including polyphenols, flavonoids, and tannins, which exhibit antioxidant, antimicrobial, and possibly pesticidal properties [6]. The recovery and use of these compounds support circular economy principles by reducing waste and driving innovation in sustainable bioproducts. Additionally, global food security faces significant challenges due to factors such as limited investment in research and infrastructure, climate change, and water scarcity [7].

The extraction of phenolic compounds and tannins from pistachio by-products was reported [8]. In addition, Zalazar et al. [9] determined the optimal conditions for polyphenol extraction with antiradical activity from PW using ethanol–water combinations, determined using the response surface method. Additionally, it has been suggested that pistachio hulls could be used to extract antioxidants, nutraceuticals, and cytoprotective compounds [10,11,12]. Anacardic, masticadienonic, and shikimic acids have shown antidiabetic, anticholinesterase, and cytotoxic activities, respectively, while anacardic acid and its derivatives have shown a selective and stronger butyrylcholinesterase (BuChE) inhibition [13]. Mokhtarpour et al. [14] suggested that pistachio by-product silage can be utilized as an alternative forage source for animals. Recent studies have highlighted the significant threat posed by phytoparasitic nematodes, which infest a wide range of crops globally. These infestations drastically reduce both yields and quality, leading to considerable economic losses. Addressing their impact remains critical for sustainable agriculture practices [15,16]. The nematode *Meloidogyne incognita* (Kofoid & White, 1919) Chitwood, 1949, attacks root systems, impairing water and nutrient uptake and reducing crop yield and quality. The estimated global economic losses caused by *M. incognita* alone amount to approximately USD 173 billion annually [17]. Traditionally, to control nematode infestation, several approaches are used such as natural enemies, enhancing cultural practices, resistant species cultivation, and/or applying pesticides; however, their widespread use has been increasingly restricted due to concerns about toxicity, environmental persistence, and negative effects on non-target organisms [18]. PW could serve as a source of bioactive molecules and provide an eco-friendly approach to managing *M. incognita* populations. Additionally, agro-industrial by-products, including PW, may play a crucial role in pest management by interacting with key enzymatic systems essential for maintaining soil health.

Cholinesterase enzymes, such as acetylcholinesterase (AChE) and BuChE, play essential roles in the neuromuscular function of various organisms, including nematodes [19]. AChE is the primary target of conventional nematicides, such as organophosphates and carbamates, used against *M. incognita* [20]. Almutairi et al. [21] reported that isolated compounds from seed cotton cake (waste) showed binding interactions targeting *M. incognita* AChE. Similarly, *Pistacia* L. genus polar extracts have demonstrated cholinesterase-inhibitory activity [22]; this mechanism may be relevant in PW applications against *M. incognita*.

Assessing the potential toxic effects of agro-industrial waste is essential for determining its environmental impact [23]. Ecotoxicological assays are essential for assessing the biological effects of waste-derived compounds. Among these, phytotoxicity tests are widely applied to study their influence on plant development, focusing primarily on seed germination and root elongation [24,25]. Since germination is a critical and environmentally sensitive phase in a plant’s life cycle, it serves as a reliable indicator of potential toxicity [26]. Therefore, this study aimed to characterize the chemical composition of *P. vera* waste decoction (PWD) with a particular focus on bioactivities that could be valorized for applications in eco-friendly agriculture.

## 2. Results

### 2.1. Chemical Profile

#### 2.1.1. Total Phenolic (TPC), Flavonoid Content (FC), and Antioxidant Activity

Total phenolic (TPC) and flavonoids content (FC) were determined by means of the Folin–Ciocalteu assay and colorimetric method with AlCl_3_, respectively. TPC was expressed as mg GAE/g dPW and FC as mg QE/g dPW (Table 1). The TPC value was 212.65 ± 21.93 mg GAE/g dPW and FC 0.022 ± 0.004 mg QE/g dPW, representing 0.01% of total phenolic compounds.

**Figure 1:**
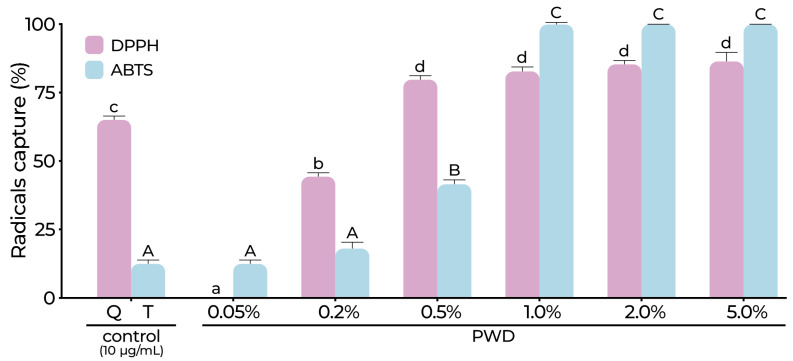
The radicals capture (DPPH and ABTS) of the PWD. The results are expressed as percentage values relative to positive control at 10 µg/mL: quercetin (Q) and Trolox (T), respectively. Statistical comparisons were conducted independently for each method. Different letters indicate significant differences between treatments within each method according to the Tukey test (*p* ≤ 0.05): lowercase letters for DPPH and uppercase letters for ABTS.

The antioxidant activity was assessed measuring the capacity to capture DPPH and ABTS radicals using dilutions of PWD (0.05, 0.2, 0.5, 1.0, 2.0, and 5.0%). DPPH showed the following values: 0.0, 44.9, 79.99, 83.58, 85.96, and 87.01%, and ABTS 12.80, 18.83, 42.01, 100.00, 100.00, and 100.00% of the capacity capture for each dilution of PWD, respectively (Figure 1). Additionally, EC_50_ values were calculated to radical DPPH (EC_50_ = 0.277) and ABTS (EC_50_ = 0.508) g/100 mL PWD. The FRAP assay revealed remarkable antioxidant capacity, reaching 7.52 mM TE/mg dPW (Table 1).

#### 2.1.2. UPLC-DAD-ESI-MS/MS Analysis

The UPLC-DAD-ESI-MS/MS analysis allowed us to obtain information about the chemical profile of the PWD. In Table 2, the peak number, retention time (RT), molecular ion [M-H^+^]^-^, main MS/MS fragments, main UV-Vis absorbance (λ) peaks, and tentative identification are presented for each compound. Twenty-six compounds were tentatively identified as follows: quinic acid (**1**), malic acid (**2**), quinic acid derivative (**4**), gallic acid (**5**), digalloyl quinic acid (**6**), protocatechuic acid (**7**),-*O*-galloyl-*β*-D-glucose (**8**), digalloyl quinic acid (**9**), tri-*O*-galloyl-glucose isomer (**10**), tetragalloyl hexose (**12**), myricetin-3-*O*-hexoside (**15**), myricetin-3-*O*-hexoside (**16**), pentagalloyl glucose isomer (**17**), quercetin galloyl hexoside (**18**), pentagalloyl glucose isomer (**20**), quercetin glucuronide (**21**), quercetin 3-*o*-rhamnoside-7-*O*-glucoside (**22**), myricetin digalloyl rhamnoside (**23**), kaempferol hexoside (**24**), quercetin (**25**), kaempferol (**26**), (16:0) anacardic acid (**27**), (13:1) anacardic acid (**28**), (13:0) anacardic acid (**29**), (15:1) anacardic acid (**30**), (17:1) and anacardic acid (**31**), while five compounds (**3**, **11**, **13**, **14**, and **19**) were not identified.

**Figure 2:**
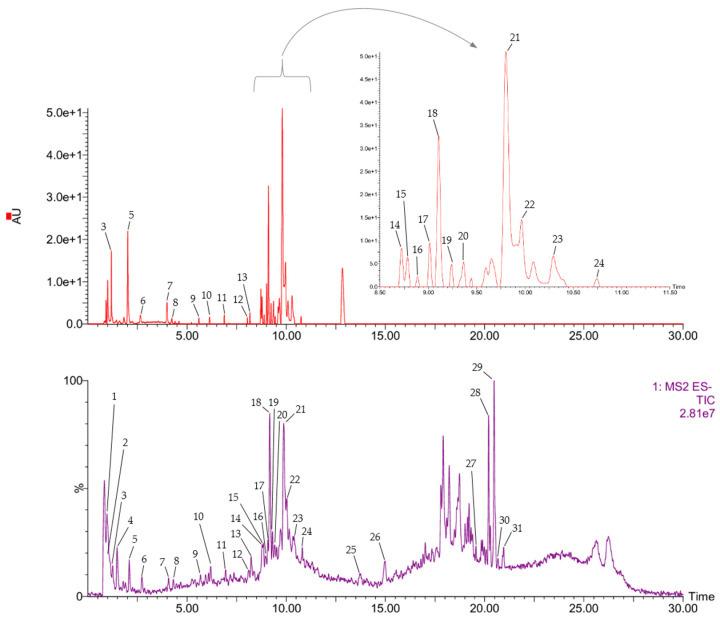
UPLC-DAD-ESI-MS/MS chromatograms (negative mode) of PWD. Peaks’ identifications are shown in Table 2.

**Figure 3:**
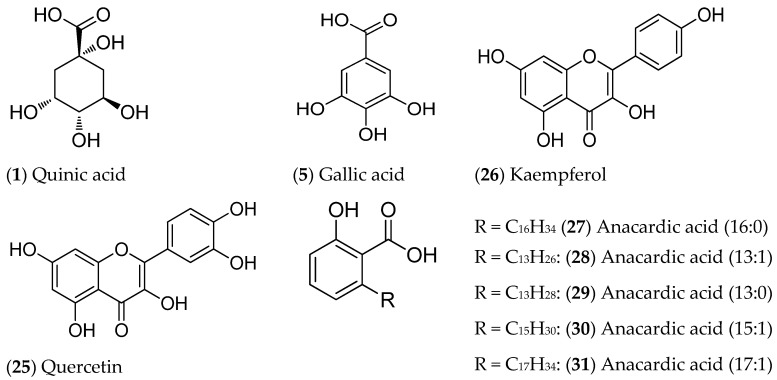
Structures of the major compounds of PWD.

The total ion current (TIC) of the ESI-MS/MS analysis in negative mode and the diode array chromatograms are shown (Figure 2). The main constituents of the PWD were tentatively identified by comparing the mass spectrometric data with the literature references and progressively numbered according to their UPLC/ESI-MS retention times. The structures of major compounds are presented in Figure 3.

### 2.2. Nematicidal and Anticholinesterases Activities

#### 2.2.1. Nematicidal Activity

**Figure 4:**
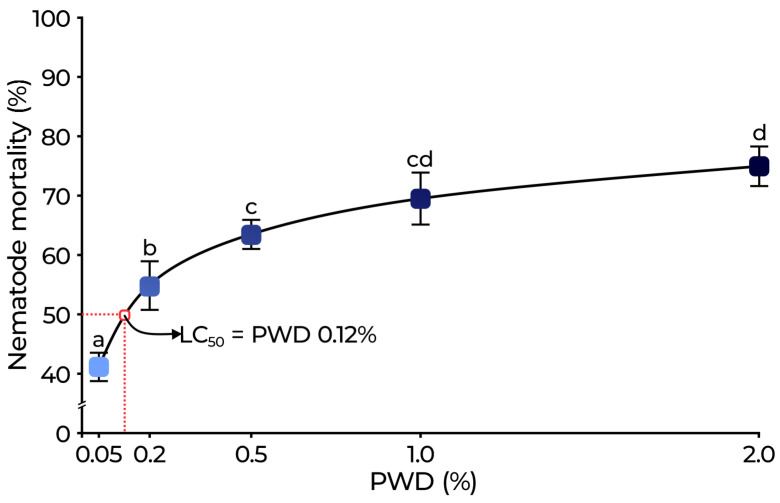
Mortality (%) of *M. incognita* exposed to PWD after 24 h. The dose-response curve was produced using CompuSyn, and the LC_50_ value for PWD was estimated at 0.12%. Different letters indicate significant differences between treatments (*p* < 0.05).

Figure 4 shows the nematicidal effect of PWD against *M. incognita*. Positive control (abamectin 0.0018%) and distilled water (negative control) were used. Mortality in the negative control (without PWD treatment) was 1.25% and was used to adjust the nematode mortality values of the PWD dilutions. Abamectin exhibited a mortality rate of 95 ± 2.73%. The results revealed a dose-dependent increase in nematode mortality, showing significant differences among treatments (*p* < 0.05). The mortality reached 41.15 ± 2.38% at 0.05% PWD and increased to 74.96 ± 3.34% at 2.0% PWD. The calculated LC_50_ value was 0.12%.

**Figure 5:**
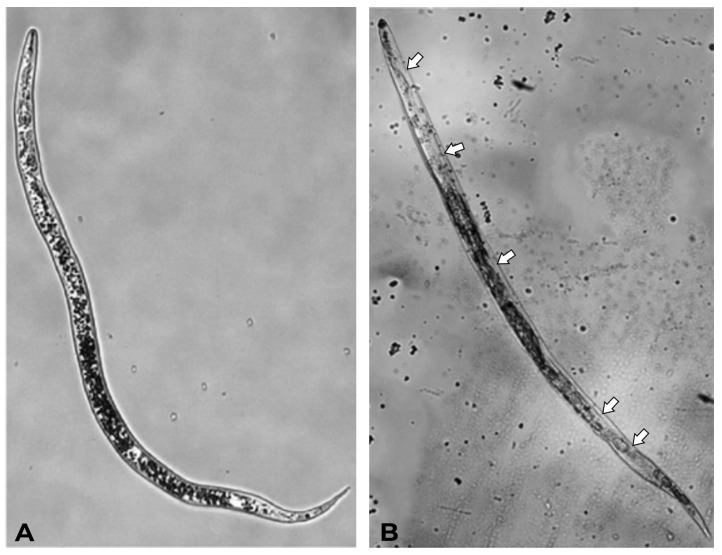
(**A**) Live *M. incognita* J2 (control). (**B**) Dead *M. incognita* J2 after 24 h of exposure to 2.0% PWD. Arrows indicate alterations in the cuticle, including disorganization and detachment from the internal tissue. Optical microscopic images (40× magnification).

After 24 h of exposure to 2.0% PWD, the morphology of dead *M. incognita* nematodes was observed (Figure 5). Microscopic examination revealed distinct structural changes between treated individuals compared to those in the control group. Figure 5A shows a live individual from the negative control preserving its characteristic phenotype. In contrast, nematodes treated with 2% PWD adopted a rigid posture, either straight or slightly bent (Figure 5B). Moreover, alterations in the cuticle were detected, including disorganization and detachment from the internal tissue, leading to a loss of its function (Figure 5B, arrows).

#### 2.2.2. Anticholinesterases Activity

In Table 3, the cholinesterase inhibitory activity of the 0.05, 0.2, 0.5, 1.0, and 2.0% PWD dilutions are shown. At 2% PWD, a remarkable inhibitory effect was detected: the inhibition rates were 42.65% for AChE and 58.90% for BuChE. In contrast, at 1% PWD, the effect was considerably lower (AChE = 12.23%, BuChE = 6.78%), while from 0.05% to 0.5% PWD, no inhibitory effect was detected. The positive control IC_50_ values for AChE and BuChE were 1.62 ± 0.05 µM and 22.07 ± 0.94 µM, respectively.

### 2.3. Phytotoxicity

**Figure 6:**
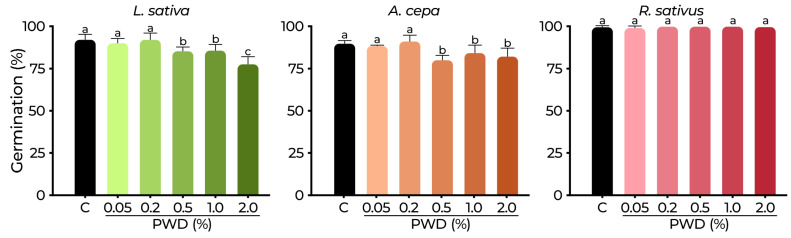
Germination percentage of *L. sativa*, *A. cepa*, and *R. sativus* exposed to PWD and control (C). Values are expressed as mean ± SD. Different letters denote significant differences between treatments (*p* < 0.05) according to the DGC test.

**Figure 7:**
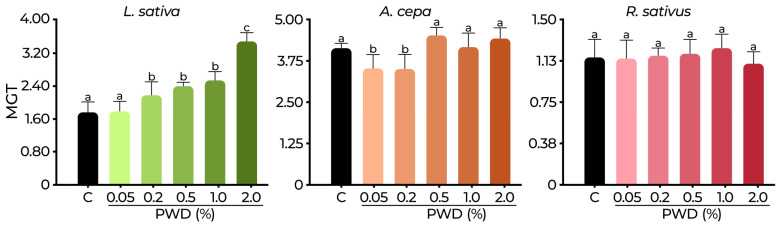
Mean germination time (MGT) of *L. sativa*, *A. cepa*, and *R. sativus* exposed to PWD and control (C). Values are expressed as mean ± SD. Different letters denote significant differences between treatments (*p* < 0.05) according to the DGC test.

The results of PWD on seed germination of *Lactuca sativa* L., *Allium cepa* L., and *Raphanus sativus* L. are summarized in Figure 6 and Figure 7. The germination percentage (GP) (Figure 6) at the control was ranged between 89% and 99.5%. The susceptibility to PWD varied among species. *R. sativus* GP was not affected in any treatment (GP = 100%) (*p* > 0.05). *L. sativa* was significatively affected by 2.0% PWD (GP = 77.5%) and GP = 85% by 0.5 and 1.0% PWD. While *A. cepa* GP values (80–84%) from 0.5% to 2.0% PWD were significatively affected compared to those in the control (*p* < 0.05), it is worth highlighting that germination remained above 78% across all treatments, emphasizing the low phytotoxicity of PWD at this developmental stage for the tested species.

The mean germination time (MGT) in *L. sativa*, *A. cepa*, and *R. sativus* are presented in Figure 7. For *L. sativa* at 2.0% PWD, the MGT was 3.48 d, representing a significant retardation compared to the control MGT of 1.74 d. This corresponds to a notable delay of 1.68 d. For *A. cepa* at 0.05 and 0.2% PWD, the MGT was 3.51 and 3.50, respectively, while the control was 4.13 d, showing a significant difference (*p* < 0.05), suggesting a stimulatory effect on germination speed due to the number of d decreasing. However, from 0.5 to 2.0% PWD, the MGT values were similar to those of the control (*p* > 0.05), ranging between 4.16 and 4.52 d. In *R. sativus,* the MGT values were similar to all treatments (*p* > 0.05), ranging from 1.10 to 1.24 d (control 1.15 d).

**Figure 8:**
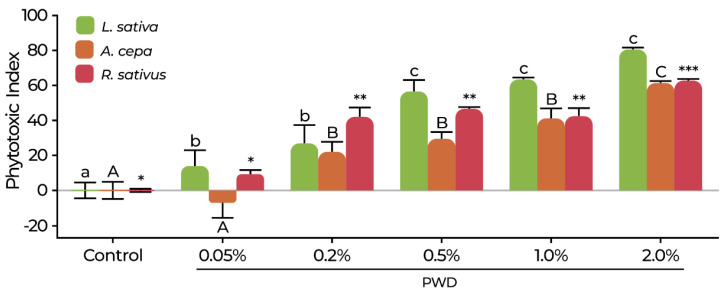
Phytotoxicity index of *L. sativa*, *A. cepa*, and *R. sativus* in response to PWD exposure. Data are presented as mean ± SD. Statistical comparisons were conducted independently for each species. Different symbols indicate significant differences between treatments within each species according to the DGC test (*p* < 0.05): lowercase letters for *L. sativa*, uppercase letters for *A. cepa*, and asterisks for *R. sativus*.

**Figure 9:**
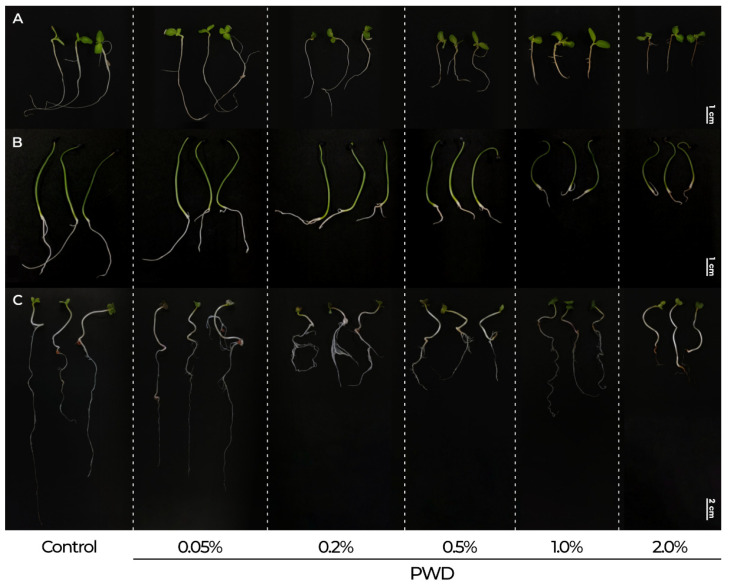
Effect on the seedling development after 10 d of exposure to PWD. (**A**) *L. sativa*, (**B**) *A. cepa*, (**C**) *R. sativum*.

The effect of PWD on seedling growth and the phytotoxicity index (PI) is presented in Figure 8 and Figure 9. The PI was significantly affected by PWD for all species tested. In *L. sativa* (Figure 8), a slight but statistically significant phytotoxic effect (*p* < 0.05) was observed at 0.05% and 0.2% PWD. A more pronounced increase in the PI was evident within the 0.5–2.0% PWD range, reaching a maximum value of 82.27 ± 5.34. Visual toxicity symptoms like intense root browning were also observed at these dilutions (Figure 9A). Regarding *A. cepa*, at 0.05% PWD, the PI (Figure 8) was slightly lower than that in the control but without statistically significant differences (*p* > 0.05). At 0.2–1.0% PWD, the PI ranged between 22.12 and 41.29, with a statistically significant difference compared to that in the control (*p* < 0.05). At 2.0% PWD, the PI reached 61.58 ± 2.72, with a statistical difference compared to those in the rest of the treatments (*p* < 0.05), confirming a dose-dependent response but with a less pronounced inhibition, although necrotic symptoms were observed at this dilution (Figure 9B). A similar tendency was observed for *R. sativus*, which at 0.05% PWD, did not show a phytotoxic effect (Figure 8). Although, in the range of 0.2–1.0% PWD, the phytotoxic index remained between 42.58 and 46.76, showing a moderate inhibitory effect. At 2.0% PWD, the PI increased significantly (*p* < 0.05) to 63.55 ± 1.58, with an evident necrotic effect (Figure 9C). In contrast to *L. sativa*, the response observed in *R. sativus* was more gradual, with no sharp increases at intermediate dilutions, indicating a higher tolerance to PWD exposure.

## 3. Discussion

Natural compounds are gaining recognition as sustainable alternatives to synthetic agrochemicals [29]. Utilizing bioactive compounds from agro-industrial by-products provides an innovative way to decrease reliance on chemical pesticides and support sustainable farming, including those reported with nematicidal properties [30]. This study focused on evaluating the nematicidal effects of PWD against *M. incognita* and examining its safety profile for horticultural crops, including *L. sativa*, *A. cepa*, and *R. sativus*. Additionally, the chemical profile was analyzed.

Phenolic compounds are recognized as the major molecules identified from *Pistacia* species. In particular, pistachio leaves were reported as a rich reservoir of phenolic compounds, including flavonoids and flavonoid glycosides [9,31,32]. The presence of gallic acid, myricetin, catechin, rutin, quercetin, gallotannin, and anacardic acid was extensively reported in leaves and hulls from pistachio [33,34,35,36]. In this work, the major compounds were identified by UPLC-DAD-ESI-MS/MS as follows: quinic acid (**1**), gallic acid (**5**), quercetin (**25**), kaempferol (**26**), and anacardic acid (**27**) (Figure 2 and Figure 3). These findings are consistent with previous studies on *P. vera* wastes; Sonmezdag et al. [37], who identified eleven phenolic compounds, found gallic acid to be the most abundant. Anacardic acid derivatives are among the major identified compounds and have been extensively reported to be present in Anacerdiaceae species nut shells. Likewise, it is recognized to present antibacterial, antitumor, anti-parasitic, anti-inflammatory, and antioxidant activities, including interactions with ROS [36].

Antioxidants, known as “free radical scavengers”, stabilize free radicals, preventing chain reactions and protecting body cells from damage, principally assigned to phenolic and flavonoid compounds. The antiradical activity of DPPH was significantly higher at PWD 0.50–5.0% dilutions ranging from 79.99 to 87.01% (Figure 1), better than that of the reference compound (Q > 65%). Although, dilutions from 1.0 to 5.0% (~100%) showed higher scavenging capacity for the ABTS radical and were significantly greater than those of the reference compound (Trolox ~12%) (Figure 1). The FRAP assay revealed a remarkable antioxidant capacity, reaching 7.52 mM TE/mg dPW (Table 1). The FRAP assay functions through electron transfer, and its efficiency is affected by the presence, quantity, and arrangement of hydroxyl groups in phenolic compounds. These phenolics can influence chemical reactions by reducing or neutralizing ROS and metal ions, such as ferric ions. The TPC was 212.65 ± 21.93 mg/g dPW and the FC was 0.022 ± 0.004 mM QE/g dPW (Table 1). These values were almost eight times higher than those reported for pistachio hull extracts by Goli et al. [10] and three times higher than those reported of the green and ripe kernels of *P. lentiscus* by Buyukkurt et al. [38]. Through an exhaustive FRAP and ORAC assay analysis, Skroza et al. [39] highlighted the relationship between phenolic content and antioxidant capacity, including gallic and quinic acid, establishing that the overall antioxidant activity is affected by the compound concentration and the number and position of functional groups. These reported data could explain the results regarding the TPC, FC, and antioxidant activity of the PWD.

Regarding nematicidal effects, phenolics and flavonoids such as *p*-hydroxybenzoic, gallic, vanillic, caffeic, and ferulic acids as well as quercetin-7-glucoside exhibited significant activity against *Meloidogyne* species [40,41,42,43,44,45]. Wuyts et al. [46] reported that kaempferol, quercetin, and myricetin repelled and inhibited *M. incognita* J2. Additionally, naringenin, hesperetin, apigenin, daidzein, and kaempferol reduced egg hatching by up to 21%. Oliveira et al. [47] further demonstrated that quercetin-7-glucoside inhibited nematode motility, reduced egg hatching, and decreased gall formation in *M. javanica*. In this work, the nematicidal assays of PWD exhibited a clear dose-dependent effect on *M. incognita* (LC_50_ = 0.12% PWD). Similarly, Hajji-Hedfi et al. [48] reported an 88% mortality rate in *M. javanica* juveniles (24 h), and egg hatching decreased significantly with the increase in concentration of *P. lentiscus* aqueous extracts. The exposure to 2.0% PWD induced significant morphological alterations in *M. incognita* J2. Abdel-Rahman et al. [49] reported that necrotic effects are likely attributable to the pro-oxidant properties of gallic acid [50]. The structural changes in the cuticle may lead to increased permeability, resulting in the loss of essential body fluids, dehydration, and eventual nematode death [51]. Additionally, cuticle disruption can weaken the nematode’s ability to protect itself from external factors such as osmotic pressure fluctuations, temperature changes, and pathogen attacks [52]. The rigid posture observed in nematodes treated with 2.0% PWD is consistent with the findings of Djiwanti et al. [53], who reported that most nematodes exposed to plant extracts assumed a rigid or slightly bent shape. *M. incognita* encodes distinct AChE isoforms essential for neuromuscular signaling and motor activity [54]. Cholinesterase inhibition leads to acetylcholine accumulation, causing continuous nerve cell stimulation, which ultimately results in the paralysis and death of nematodes [55]. The best cholinesterase inhibition results occurred at 1.0–2.0% PWD with a J2 mortality >65%, suggesting that cholinergic interference may contribute to nematode lethality. Several phenolic compounds have been reported to modulate enzyme activity [56]. Alsharif et al. [57] demonstrated a strong inhibitory effect on AChE, likely attributed to the phenolic content of the extract pistachio. These findings highlight the relevance of enzymatic disruption as an additional factor contributing to the nematicidal action of PWD.

Based on the LC_50_ for nematicidal activity, the GP and MGT results of PWD against *L. sativa*, *A. cepa*, and *R. sativus* suggest that PWD at 0.12%, effective for nematode control, does not substantially inhibit germination, indicating a selective nematicide effect in PWD. According to Römheld [58], the relationship between nutrient concentration and plant growth could be examined, along with the visual symptoms to help diagnose toxicity. *L. sativa* showed the greatest sensitivity: with a significant increase from 0.05% PWD, root browning and necrotic effects were visually detected at 2.0% PWD, a value 16.66 times higher than that of the nematicidal LC_50_. Previous research indicates that elevated levels of phenolic compounds, such as *p*-coumaric, caffeic, and ferulic acids, can trigger necrosis due to their pro-oxidant activity [59]. Notably, a nematicidal LC_50_ of 0.12% is significantly lower than that in the dilutions associated with phytotoxic effects, emphasizing a wide safety margin suitable for agricultural use. Research on *Pistacia* species has largely focused on their allelopathic properties as potential natural herbicides. Pot experiments have shown their effectiveness against weeds like *Diplotaxis erucoides*, *Sonchus arvensis*, and *Papaver hybridum* [60]. Pistachio hulls and leaves have also been observed to suppress the germination and growth of various weed species, including *Amaranthus retroflexus*, *Rumex acetosa*, *Echium vulgare*, *Scabiosa triandra*, *Taraxacum officinale*, and *Sinapis arvensis* [33,60,61]. The allelopathic strength, however, varies across species, with *P. terebinthus* and *P. lentiscus* displaying greater inhibitory effects on weeds like *Echium plantagineum* and *Lavandula stoechas* [34]. This study shifts attention from herbicidal uses to examining safe dilution thresholds of PWD for horticultural crops. It aims to identify dose ranges that successfully manage nematodes while supporting the healthy growth of *L. sativa*, *A. cepa*, and *R. sativus*. The findings offer valuable perspectives on utilizing PWD as a natural nematicide while preserving crop productivity.

## 4. Materials and Methods

### 4.1. Chemicals

Ultrapure water (<5 μg/LTOC) was from a water purification system Arium 12661316-RO, plus an Arium 611 UVunit (Sartorius, Göttingen, Germany). Commercial Folin–Ciocalteu reagent, 1,1 Diphenyl-2-picrylhydrazyl (DPPH), ferric chloride hexahydrate, aluminum trichloride, gallic acid (GA), quercetin (Q), ABTS (2,20-azino-bis-(3-ethylbenz-thiazoline-6-sulfonic acid), Trolox (6-hydroxy 2,5,7,8-tetramethylchroman-2-carboxylic acid), abamectin (Abamex^®^, San Diego, CA, USA), potassium phosphate (K_2_HPO_4_), sodium dihydrogen phosphate (NaH_2_PO_4_), sodium Chloride (NaCl), 5,5′-dithio-bis-(2-nitrobenzoic acid) (DTNB), acetylthiocholine iodide (ATC), butyrylthiocholine iodide (BTC), Galantamine (Gal), AChE from *Electrophorus electricus* (electric eel), and BuChE from equine serum were purchased from Sigma-Aldrich (Buenos Aires, Argentina).

### 4.2. Samples and Extraction Procedure

*Samples*: pistachio wastes were provided by Pisté SRL from San Juan, Argentina. *Extraction procedure*: pistachio waste (PW) samples were oven-dried at 60 °C until reaching constant weight. Subsequently, a pooled sample (150 g) of dried pistachio waste (dPW) was boiled for 15 min in distilled water (1.5 L), cooled, then filtered with a Whatman paper filter, N° 4, and autoclaved for 20 min at 121 °C; the final volume was adjusted to 10% *w*/*v* and named as pistachio waste decoction (PWD), and for bioassays, the dilutions of 2.0, 1.0, 0.5, 0.2, and 0.05% *v*/*v* were prepared.

### 4.3. UPLC-DAD-ESI-MS/MS Analysis

The analysis was performed in an ACQUITY H–Class UPLC instrument equipped with an Acquity UPLC PDA eLambda detector and a XEVO TQ-S micro triple quadrupole mass spectrometer (Waters Corp., Milford, MA, USA). An UPLC ACQUITY BEH C18 (1.7 µm, 2.1 mm × 100 mm) column was used for separation at 35 °C. The mobile phase consisted of A (1% formic acid) and B (methanol, 1% formic acid) with a flow rate of 0.3 mL/min. The gradient conditions were as follows: initially, 98% A and hold for 1 min; at 8 min, 63% A; at 13.20 min, 60% A; at 18 min, 0% A and hold for 6 min; at 25 min, 98% A, and hold for 5 min, complete in 30 min. The PWD sample was dissolved in a mixture of methanol/water (50:50) with 1% formic acid and filtered through a membrane filter (0.22 µm). The injection volume was 5 µL. The UV/Vis absorption spectra were recorded in the range of 210–700 nm. The capillary, cone, and collision energies were 3 kV, 44 V, and 20 eV, respectively. The data were acquired in ESI negative mode, MS2 scan function (60–1000 Da), and processed using MassLynx Software V4.2 (Waters, Milford, MA, USA).

### 4.4. Assessment of Total Phenolic Content (TPC) and Flavonoid Content (FC)

*Total Phenolic Content*. TPC was determined according to Gimenez Guerrero et al. [62]. In a 96-well microplate, 10 μL of PWD dilutions, 12.5 μL of diluted Folin–Ciocalteu reagent, 37.5 μL of 20% (*w*/*v*) Na_2_CO_3_ were added and incubated for 30 min at 25 °C in the dark. The absorbance was read at 750 nm using a Multiskan FC microplate reader (Thermo Scientific, Waltham, MA, USA). The calibration curve was constructed using GA at concentrations of 0, 0.15, 0.3, 0.6, 1.2, and 2.35 mM. Results were expressed as mg of GA equivalents per gram of dPW (mg GAE/g dPW).

*Flavonoid Content*. The aluminum trichloride (AlCl_3_) colorimetric method was employed to assess the FC according to Ismail et al. [63] with modifications. In each well, 125 μL of PWD dilutions and 125 μL of 2% (*w*/*v*) AlCl_3_ were added. The solution was incubated for 10 min at 25 °C. The absorbance was measured at 450 nm using a microplate reader (Thermo Scientific, Waltham, MA, USA). The calibration curve was constructed using Q standard solutions at concentrations of 0.00, 0.03, 0.07, 0.15, 0.22, and 0.30 mM. Results were expressed as mM of Q equivalents per gram of dPW (mg QE/g dPW). All the values were obtained in triplicate (TPC and FC), and are reported as mean ± standard deviation (SD).

### 4.5. Antioxidant Activity (DPPH, ABTS, and FRAP Assays)

*DPPH Radical Scavenging Activity*: PWD dilutions were assayed according to Gimenez-Guerrero et al. [62] with modifications and reacted. The absorbance was measured at 517 nm using a Multiskan FC microplate reader (Thermo Scientific, Waltham, MA, USA). The percentage of the capture was calculated using the following formula:

DPPH scavenging capacity (%) = [1 − ((A_S_ − A_C_)/(A_DPPH_)] × 100 (1)

where A_s_ is the sample absorbance; A_c_ is the control absorbance; and A_DPPH_ is the DPPH absorbance.

The scavenging capacity percentage values including EC_50_ were calculated as the mean standard deviation of three independent assays. Q was used as the reference compound.

*ABTS Radical Scavenging Activity*: the method described by Re et al. [64] was used. Briefly, 10 μL of PWD dilutions or Trolox standard was mixed with 200 μL of ABTS^+^ (dissolved in PBS) and incubated for 4 min at 30 °C, and the absorbance was measured at 734 nm using a Multiskan FC microplate reader (Thermo Scientific, Waltham, MA, USA). The scavenging percentage was calculated with the same formula used for DPPH. The PWD dilutions providing 50% of radical scavenging activity (EC_50_) were calculated by plotting the inhibition percentage (A_734_) against the PWD. All measures were performed in triplicate.

*Ferric-Reducing Antioxidant Power Assay (FRAP)*: this assay was performed according to Gimenez-Guerrero [63], with modifications. Ten µL of PWD dilution was mixed with 190 µL of FRAP reagent (300 mM acetate buffer at pH 3.6, 10 mM 2,4,6-tripyridyl-s-triazine dissolved in 40 mM HCl, and 20 mM FeCl_3_·6H_2_O in a 10:1:1 ratio). After 30 min of incubation at 25 °C, absorbance was measured at 595 nm using a Multiskan FC microplate reader (Thermo Scientific, Waltham, MA, USA). A calibration curve was generated using Trolox (0–1 mM), and results were expressed as the mM of Trolox equivalents per gram of dried pistachio waste (mM TE/g dPW).

### 4.6. Nematicidal Activity

Populations of *M. incognita* were isolated from a susceptible tomato cultivar (*Solanum lycopersicum* L.) collected in San Juan, Argentina. The nematodes were then reared under laboratory conditions using tomato seedlings grown in pots. After 40 d, infected roots were collected and carefully washed with tap water. The egg masses were manually extracted under a stereoscopic microscope (1.6×) and immersed in a 1% NaOCl solution for 4 min to dissolve the gelatinous matrix. The eggs were then incubated in a growth chamber at 28 ± 1 °C. After hatching, second-stage juveniles (J2) were collected for up to 3 d [65]. The bioassays were conducted in 50 mm glass Petri dishes, and 20 nematodes J2 were placed in 10 mL of PWD dilutions (0.05, 0.2, 0.5, 1.0, and 2.0%). Distilled water was used as a negative control, while abamectin (0.0018% *v*/*v*) served as a positive control. Each treatment was conducted in five replicates. Petri dishes were incubated in the dark at 28 °C for 24 h, after which inactive individuals were counted. The mortality was confirmed when the inactive juveniles remained unresponsive after being transferred to distilled water [65]. The natural mortality observed in the negative control was considered, and the mortality percentage was calculated applying the Schneider–Orelli formula:

NM% = (MT% − MCO%)/(100 − MCO%) × 100(2)

where NM% is the nematode mortality percentage; MT% is the mortality percentage in the treatments; and MCO% is the mortality percentage in the negative control.

Data were analyzed using a one-way ANOVA, and treatment means were compared using Fisher’s LSD test. Statistical analyses were performed using InfoStat [66]. The median lethal concentration (LC_50_) was determined using the CompuSyn software, which applies the Chou-Talalay method for dose-response analysis based on the median-effect principle [67].

### 4.7. Cholinesterase Inhibitory Activities

PWD were centrifuged at 3000 rpm for 10 min to remove suspended particles, using a Rolco CM 2036 centrifuge (Buenos Aires, Argentina). The inhibitory activity was assessed according to the Ellman method [68] with modifications. In a 96-well microplate, 50 µL of AChE or BuChE solution (0.25 U/mL in PBS: 8 mM K_2_HPO_4_, 2.3 mM NaH_2_PO_4_, 0.15 M NaCl, pH 7.5) was incubated with 50 µL of PWD dilution at 25 °C for 30 min. Then, 100 µL of the substrate solution, containing DTNB (0.3 mM) and ATC or BTC (0.6 mM), prepared in a saline solution with Na_2_HPO_4_ (pH 7.5), was added. After 5 min, the absorbance at 405 nm was recorded using a Multiskan FC microplate reader (Thermo Scientific, Waltham, MA, USA). Gal was used as a positive control, and PBS as a negative control. The enzyme inhibition percentage (%I) was calculated as follows:

%I = 100 − (S-Bs/C-Bc) × 100(3)

where S is sample absorbance, Bs is sample blank absorbance, C is negative control absorbance, and Bc is control blank absorbance

The IC_50_ values were determined by fitting the dose-response curve using GraphPad Prism 10.4.1 (GraphPad Inc., San Diego, CA, USA).

### 4.8. Phytotoxic Activity

Healthy crop seeds of *L. sativa* (lettuce), *A. cepa* (onion), and *R. sativus* (radish) were selected for the germination test. To prevent fungal or bacterial contamination, the seeds were surface-sterilized by immersion in a 1% NaOCl solution for 5 min. After sterilization, the seeds were thoroughly washed three times with deionized water. Each treatment consisted of four replicates of 25 seeds per species, placed on filter paper in 9 cm diameter Petri dishes (FILTER-LAB^®^). The filter papers were moistened with 3 mL of the PWD dilutions (0.05, 0.2, 0.5, 1.0, and 2.0%), while control dishes were moistened with 3 mL of deionized water. The Petri dishes were then incubated in a growth chamber (INGELAB, Buenos Aires, Argentina) under a 12 h light/12 h dark cycle at a constant temperature of 25 °C. Petri dishes were randomly distributed within the chamber, changing their positions daily to minimize positional effects. The number of germinated seeds was recorded every 24 h for 10 d. Germination was considered complete when the emerging radicle reached approximately ≥1 mm in length. The germination percentage (GP), mean germination time (MGT), and phytotoxicity index (PI) were calculated based on seed germination counts and root length measurements, as described below. Raw data are provided in Supplementary Materials (Table S1). The final GP was calculated as the number of germinated seeds/total number of seeds × 100 [69]. The MTG was estimated using the following formula:

MTG = ∑ (n × d)/N(4)

where n is the number of seeds germinated between scoring intervals, d is the incubation period in d at that time point, and N is the total number of seeds germinated in the treatment.

After 10 d of growth, the impact of PWD treatment on the root was evaluated by measuring the root length (RL) using a stainless-steel digital caliper (Wemberley, accuracy ±0.01 mm) [70]. The PI was then calculated using the following formula:

PI = 1 − (RLt/RLc) × 100(5)

where RLt is the root length in the PWD-treated group and RLc is the root length in the control group.

## 5. Conclusions

This study highlights the potential of *Pistacia vera* waste as a biocidal agent for managing *M. incognita*. The findings strongly suggest that the nematicidal potential of PWD is likely associated with high levels of phenolic compounds, such as gallic and anacardic acids, which act as key contributors, along with antioxidant and anticholinesterase activities. PWD could serve as a key component in developing a novel biopesticide with an integrated approach, representing a more promising strategy for nematode control. Advancing the understanding of interactions between the compounds and nematode physiology could serve as a starting point for the development of sustainable, plant-based nematicides, offering an environmentally friendly alternative to conventional chemical control strategies. This study encourages further research including the mechanisms of action and the interaction of phenolic compounds in the soil, thereby adding value to pistachio residues. Field trials are essential for assessing its effectiveness under agricultural practice.

Additionally, PWD demonstrated strong radical scavenging potential, making it an excellent candidate for isolating antioxidant compounds with applications in nutraceuticals, cytoprotective agents, and cosmetic formulations. Furthermore, the identification of anacardic acids, recognized for its antidiabetic properties and selective BuChE inhibitory activity, opens up opportunities for in-depth research with these wastes, aiming to enhance its value and explore its potential for sustainable and innovative uses.

## Figures and Tables

**Table 1:** Total phenolic and flavonoid content and antiradical activity (FRAP).

TPC ^a^	FC ^b^	FRAP ^c^
(mg GAE/g dPW)	(mg QE/g dPW)	(mM TE/g dPW)
212.65 ± 21.93	0.022 ± 0.004	7.52 ± 0.19

^a^ Total phenolic content (TPC) expressed as mg gallic acid (GA) equivalent/g of dried pistachio waste (dPW). ^b^ Flavonoid content (FC) expressed as mg quercetin (Q) equivalent/g of dried pistachio waste (dPW). ^c^ Ferric-reducing antioxidant power (FRAP) expressed as mM Trolox equivalent/g of dried pistachio waste (dPW). All results are presented as mean ± SD.

**Table 2:** UPLC-DAD-ESI-MS/MS analysis of PWD.

Peak	RT (min)	[M-H^+^]^−^	ESI-MS Fragmentation	UV-Vis (λ)	Tentative Identification	Ref.
**1**	0.97	191(100)	171(22), 127(12), 85(10)	n.d.	Quinic acid	[5,27]
**2**	1.08	133(100)	71(61), 115(50), 89(10)	n.d.	Malic acid	[5,27]
**3**	1.26	171(100)	109(60), 127(58)	239	Unidentified	[5,27]
**4**	1.48	427(45)	111(100), 191(90), 87(85)	n.d.	Quinic acid derivative	[28]
**5**	2.09	169(100)	125(98), 97(20), 107(6)	271	Gallic acid	[28]
**6**	2.71	495(5)	191(100), 343(25)	274	Digalloyl quinic acid	[27,28]
**7**	4.07	153(40)	109(100), 125(5)	260	Protocatechuic acid	[27]
**8**	4.30	331(52)	169(100), 125(15)	260	1-*O*-galloyl-β-D-glucose	[27]
**9**	5.71	495(35)	343(100), 191(50)	277	Digalloyl quinic acid	[27,28]
**10**	6.17	635(30)	423(100), 465(12), 483(5)	279	Tri-*O*-galloyl-glucose isomer	[5]
**11**	6.95	483(10)	163(100), 325(30), 199(18)	282	Unidentified	
**12**	8.12	787(100)	465(15), 635(10), 617(5)	276	Tetragalloyl hexose	[5,27]
**13**	8.24	787(75)	631(100), 463(10)	272, 359	Unidentified	
**14**	8.78	635(10)	317(100), 493(52), 515(30)	264, 300, 357	Unidentified	
**15**	8.85	479(100)	316(28), 392(8)	266, 292, 358	Myricetin-3-*O*-hexoside	[28]
**16**	8.94	625(18)	479(100), 316(23)	266, 290, 357	Myricetin-3-*O*-hexoside	[28]
**17**	9.08	939(70)	469(100), 169(40), 625(29)	280	Pentagalloyl glucose isomer	[5,27]
**18**	9.16	615(100)	463(6), 313(4), 301(2)	264, 290, 354	Quercetin galloyl hexoside	[5,6,7,8,9,10,11,12,13,14,15,16,17,18,19,20,21,22,23,24,25,26,27,28]
**19**	9.31	615(100)	393(12), 463(8), 217(5)	267, 356	Unidentified	
**20**	9.44	939(15)	469(100), 393(16), 615(15)	279	Pentagalloyl glucose isomer	[5,27]
**21**	9.87	477(60)	301(100)	257, 355	Quercetin glucuronide	[28]
**22**	10.04	609(20)	463(100), 301(60), 545(12)	266, 355	Quercetin 3-*O*-rhamnoside-7-*O*-glucoside	[5,28]
**23**	10.39	767(100)	263(50), 463(12), 615(5)	286, 398	Myricetin digalloyl rhamnoside	[28]
**24**	10.81	447(100)	285(20), 284(18), 151(15)	269, 355	Kaempferol hexoside	[27,28]
**25**	13.74	301(100)	151(80), 217(25), 179(20)	n.d.	Quercetin	[5]
**26**	14.98	285(100)	199(5), 151(5), 175(5)	n.d.	Kaempferol	[5]
**27**	19.56	361(100)	299(25), 219(20), 317(20)	n.d.	(16:0) Anacardic acid	[5,27]
**28**	20.20	317(70)	273(100)	n.d.	(13:1) Anacardic acid	[5,27]
**29**	20.47	319(55)	275(100)	n.d.	(13:0) Anacardic acid	[5,27]
**30**	20.52	345(80)	301(100)	n.d.	(15:1) Anacardic acid	[5,27]
**31**	20.95	373(80)	329(100)	n.d.	(17:1) Anacardic acid	[5,27]

n.d.: not detected.

**Table 3:** Cholinesterase inhibitory activities of PWD.

PWD (%)	% Inhibitory (Mean ± SD)	
	AChE	BuChE
0.05	nd	nd
0.2	nd	nd
0.5	nd	nd
1.0	12.23 ± 1.27	6.78 ± 1.11
2.0	42.65 ± 2.15	58.90 ± 1.07

nd: not detected. Results are presented as mean ± SD.

## Data Availability

The original contributions presented in this study are included in the article. Further inquiries can be directed to the corresponding author.

## Supplementary Materials

The following supporting information can be downloaded at: https://www.mdpi.com/article/10.3390/plants14101420/s1, Table S1. Raw data of seed germination and root length for *Allium cepa*, *Lactuca sativa*, and *Raphanus sativus* after exposure to *Pistacia vera* waste decoction (PWD).
