# Supplementary material for: Agro-Industrial Waste from Pistacia vera: Chemical Profile and Bioactive Properties
Source: Plants (Basel). 2025 May 9;14(10):1420. doi: 10.3390/plants14101420 (PMC12115079; doi:10.3390/plants14101420)
Supplement: Supplementary file 1 [file plants-14-01420-s001.zip › plants-3598577-supplementary.pdf]

# Agro-industrial Waste from *Pistacia vera*: Chemical Profile and Bioactive Properties

**Mauricio Piñeiro [1,2], Victoria Parera [1], Javier E. Ortiz [1], Olimpia Llalla-Cordova [2], Sofia Manrique [1], Brisa Castro [1], Maximiliano Ighani [3], Lorena C. Luna [1§] and Gabriela E. Feresin [1,2§]**

## Supplementary Materials

**Table S1.** Raw data of seed germination and root length for *Allium cepa*, *Lactuca sativa,* and *Raphanus sativus* after exposure to *Pistacia vera* waste decoction (PWD).

*A. cepa*

| | | seed germination | | | | | | | | | |
|---|---|---|---|---|---|---|---|---|---|---|---|
| treatment | repetition | 1 | 2 | 3 | 4 | 5 | 6 | 7 | 8 | 9 | 10 |
| control | 1 | 0 | 1 | 8 | 19 | 19 | 20 | 21 | 22 | 22 | 22 |
| control | 2 | 0 | 1 | 6 | 17 | 19 | 19 | 21 | 21 | 21 | 21 |
| control | 3 | 0 | 2 | 15 | 15 | 17 | 17 | 20 | 20 | 21 | 23 |
| control | 4 | 0 | 1 | 7 | 10 | 15 | 19 | 20 | 20 | 23 | 24 |
| 2% | 1 | 0 | 1 | 7 | 11 | 12 | 13 | 14 | 17 | 18 | 19 |
| 2% | 2 | 0 | 4 | 9 | 13 | 15 | 17 | 18 | 18 | 19 | 20 |
| 2% | 3 | 0 | 3 | 8 | 12 | 17 | 20 | 21 | 23 | 23 | 22 |
| 2% | 4 | 0 | 2 | 7 | 11 | 13 | 15 | 19 | 19 | 20 | 20 |
| 1% | 1 | 0 | 5 | 9 | 13 | 15 | 17 | 19 | 19 | 20 | 21 |
| 1% | 2 | 0 | 1 | 10 | 15 | 16 | 17 | 19 | 19 | 21 | 21 |
| 1% | 3 | 0 | 2 | 9 | 14 | 16 | 16 | 17 | 19 | 22 | 22 |
| 1% | 4 | 0 | 2 | 13 | 18 | 18 | 18 | 18 | 19 | 19 | 20 |
| 0,50% | 1 | 0 | 3 | 6 | 12 | 12 | 13 | 15 | 18 | 18 | 18 |
| 0,50% | 2 | 0 | 6 | 12 | 15 | 16 | 18 | 18 | 18 | 19 | 19 |
| 0,50% | 3 | 0 | 3 | 8 | 16 | 18 | 20 | 21 | 23 | 23 | 23 |
| 0,50% | 4 | 0 | 2 | 7 | 11 | 13 | 15 | 19 | 19 | 20 | 20 |
| 0,20% | 1 | 0 | 4 | 12 | 16 | 18 | 20 | 21 | 21 | 22 | 22 |
| 0,20% | 2 | 0 | 4 | 12 | 17 | 20 | 21 | 22 | 23 | 23 | 23 |
| 0,20% | 3 | 0 | 7 | 21 | 22 | 23 | 24 | 24 | 24 | 24 | 24 |
| 0,20% | 4 | 0 | 4 | 19 | 19 | 20 | 20 | 21 | 22 | 22 | 22 |
| 0,05% | 1 | 0 | 10 | 20 | 20 | 20 | 20 | 22 | 22 | 22 | 22 |
| 0,05% | 2 | 0 | 5 | 11 | 16 | 16 | 17 | 20 | 21 | 21 | 21 |
| 0,05% | 3 | 0 | 7 | 11 | 16 | 17 | 19 | 20 | 22 | 22 | 23 |
| 0,05% | 4 | 0 | 6 | 18 | 21 | 21 | 21 | 21 | 21 | 21 | 21 |

| | | root lenght | | | | | | | | | |
|---|---|---|---|---|---|---|---|---|---|---|---|
| treatment | repetition | 1 | 2 | 3 | 4 | 5 | 6 | 7 | 8 | 9 | 10 |
| control | 1 | 2,1 | 1,3 | 1,5 | 0,8 | 1,1 | 1,6 | 1,3 | 1,6 | 3,4 | 1,3 |
| control | 2 | 1,2 | 3,8 | 4 | 4,5 | 1,1 | 3,7 | 2,8 | 2,1 | 2 | 0,8 |
| control | 3 | 3,4 | 2,3 | 2,2 | 1,6 | 1,4 | 4,1 | 1,4 | 1,8 | 1,7 | 1,7 |
| control | 4 | 1,4 | 2,6 | 1,2 | 4,3 | 2,3 | 3,1 | 4 | 2,2 | 3,6 | 2 |
| 2% | 1 | 1,2 | 1,3 | 0,5 | 0,9 | 0,6 | 0,6 | 0,8 | 0,3 | 0,3 | 0,4 |
| 2% | 2 | 0,3 | 1 | 0,8 | 0,8 | 1,2 | 1,2 | 0,7 | 1,1 | 1 | 0,8 |

| | | | | | | | | | | | |
|---|---|---|---|---|---|---|---|---|---|---|---|
| 2% | 3 | 1,3 | 0,9 | 1,1 | 1,1 | 1,1 | 0,8 | 0,9 | 0,8 | 0,9 | 0,8 |
| 2% | 4 | 1,2 | 0,9 | 1,1 | 0,9 | 0,8 | 0,7 | 0,6 | 0,7 | 1 | 1,1 |
| 1% | 1 | 1,1 | 1,5 | 1,4 | 0,9 | 1,2 | 0,9 | 1,1 | 0,8 | 0,9 | 1,2 |
| 1% | 2 | 1,3 | 1,3 | 0,6 | 0,7 | 1,1 | 1,5 | 1,1 | 0,9 | 1,3 | 1 |
| 1% | 3 | 1 | 1,9 | 1,4 | 2 | 1,3 | 1,2 | 0,6 | 1,3 | 1,2 | 1,3 |
| 1% | 4 | 1,8 | 2,3 | 2,1 | 1,2 | 2,1 | 1 | 0,9 | 1,5 | 1,7 | 1,1 |
| 0,50% | 1 | 2,8 | 1,7 | 1 | 2 | 2,3 | 1,1 | 0,5 | 1,5 | 1,2 | 1,5 |
| 0,50% | 2 | 1,8 | 1,3 | 1,5 | 1,7 | 1,6 | 1,4 | 1,2 | 1,7 | 1,3 | 1,2 |
| 0,50% | 3 | 1 | 2,4 | 2,1 | 1 | 1,1 | 2,2 | 1,9 | 1,4 | 1,4 | 1,6 |
| 0,50% | 4 | 1,2 | 1,8 | 1,9 | 1,8 | 1,5 | 1 | 1,1 | 1,2 | 1,3 | 2,2 |
| 0,20% | 1 | 2,2 | 1,9 | 2 | 2,1 | 2,8 | 2,4 | 1,6 | 1,2 | 1,5 | 0,8 |
| 0,20% | 2 | 1,5 | 1,6 | 1,9 | 1,1 | 1,5 | 2,3 | 1,4 | 1,3 | 2,1 | 1,2 |
| 0,20% | 3 | 1,8 | 2,9 | 2,1 | 2 | 1,5 | 2,4 | 1,6 | 1,7 | 2 | 1,7 |
| 0,20% | 4 | 2,1 | 1,7 | 1,9 | 1,7 | 1,6 | 1,8 | 1,3 | 1,2 | 1,4 | 1 |
| 0,05% | 1 | 2,2 | 2,4 | 2,8 | 2,2 | 1,4 | 2,1 | 2,8 | 1 | 1,1 | 2,1 |
| 0,05% | 2 | 5,9 | 2,6 | 3,5 | 2,5 | 2,3 | 2,2 | 2,9 | 1,2 | 1,5 | 2 |
| 0,05% | 3 | 2,1 | 2,1 | 1,4 | 1,1 | 1,6 | 2,3 | 1,5 | 0,8 | 1,1 | 0,6 |
| 0,05% | 4 | 3,8 | 3,3 | 4,3 | 4 | 4,8 | 3,6 | 2,8 | 4 | 2,2 | 2,5 |

*L. sativa*

| | | seed germination | | | | | | | | | |
|---|---|---|---|---|---|---|---|---|---|---|---|
| treatment | repetition | 1 | 2 | 3 | 4 | 5 | 6 | 7 | 8 | 9 | 10 |
| control | 1 | 3 | 13 | 17 | 21 | 21 | 21 | 21 | 22 | 22 | 22 |
| control | 2 | 12 | 20 | 22 | 22 | 23 | 23 | 23 | 23 | 23 | 23 |
| control | 3 | 16 | 20 | 23 | 23 | 23 | 23 | 23 | 23 | 23 | 23 |
| control | 4 | 2 | 13 | 21 | 24 | 24 | 24 | 24 | 24 | 24 | 24 |
| 2% | 1 | 1 | 3 | 13 | 15 | 17 | 18 | 18 | 19 | 19 | 19 |
| 2% | 2 | 1 | 5 | 18 | 18 | 18 | 18 | 18 | 20 | 20 | 20 |
| 2% | 3 | 1 | 4 | 10 | 14 | 16 | 18 | 19 | 19 | 19 | 19 |
| 2% | 4 | 0 | 4 | 8 | 18 | 18 | 20 | 20 | 20 | 20 | 20 |
| 1% | 1 | 2 | 12 | 15 | 15 | 17 | 19 | 20 | 20 | 20 | 20 |
| 1% | 2 | 5 | 9 | 10 | 13 | 18 | 18 | 19 | 21 | 22 | 22 |
| 1% | 3 | 5 | 13 | 18 | 19 | 19 | 20 | 20 | 20 | 20 | 20 |
| 1% | 4 | 2 | 12 | 18 | 22 | 22 | 24 | 23 | 23 | 23 | 23 |
| 0,50% | 1 | 6 | 11 | 20 | 20 | 20 | 20 | 20 | 20 | 20 | 20 |
| 0,50% | 2 | 6 | 12 | 18 | 20 | 20 | 20 | 20 | 20 | 20 | 20 |
| 0,50% | 3 | 4 | 13 | 17 | 22 | 22 | 22 | 22 | 22 | 22 | 22 |
| 0,50% | 4 | 1 | 19 | 21 | 23 | 23 | 23 | 23 | 23 | 23 | 23 |
| 0,20% | 1 | 9 | 12 | 22 | 22 | 22 | 22 | 24 | 24 | 24 | 24 |
| 0,20% | 2 | 5 | 12 | 20 | 21 | 21 | 22 | 23 | 23 | 23 | 23 |
| 0,20% | 3 | 8 | 14 | 18 | 20 | 21 | 21 | 22 | 22 | 22 | 22 |
| 0,20% | 4 | 12 | 16 | 19 | 19 | 21 | 23 | 23 | 23 | 23 | 23 |
| 0,05% | 1 | 11 | 18 | 24 | 23 | 23 | 23 | 23 | 23 | 23 | 23 |
| 0,05% | 2 | 11 | 23 | 23 | 23 | 23 | 23 | 23 | 23 | 23 | 23 |
| 0,05% | 3 | 7 | 15 | 16 | 19 | 21 | 21 | 21 | 21 | 21 | 21 |
| 0,05% | 4 | 10 | 16 | 21 | 21 | 21 | 23 | 23 | 23 | 23 | 23 |

| | | root lenght | | | | | | | | | |
|---|---|---|---|---|---|---|---|---|---|---|---|
| treatment | repetition | 1 | 2 | 3 | 4 | 5 | 6 | 7 | 8 | 9 | 10 |
| control | 1 | 6,8 | 6,5 | 7,9 | 9,5 | 7,1 | 7,3 | 6,4 | 9,4 | 9,8 | 4,5 |
| control | 2 | 8,3 | 9,3 | 9,5 | 9,5 | 7,8 | 8,4 | 10,2 | 8,2 | 9,7 | 10 |
| control | 3 | 5,6 | 8,8 | 9 | 9,3 | 10,2 | 9,8 | 8,1 | 7,5 | 8,2 | 9,3 |
| control | 4 | 6,2 | 7,4 | 8,3 | 7,1 | 9,2 | 8,4 | 6,9 | 8 | 8,6 | 10,3 |
| 2% | 1 | 1,5 | 2,4 | 2 | 2,6 | 2,7 | 1,8 | 2,7 | 1,9 | 1,2 | 2,7 |
| 2% | 2 | 1,3 | 1,7 | 1,6 | 1,4 | 1,1 | 0,7 | 0,9 | 1,3 | 1,3 | 1,2 |
| 2% | 3 | 1,9 | 2,3 | 1 | 2,1 | 1,2 | 1,2 | 1 | 1,2 | 1,2 | 0,7 |
| 2% | 4 | 1,4 | 1,2 | 1,3 | 2,5 | 1,2 | 2,2 | 0,4 | 0,1 | 0,3 | 0,4 |
| 1% | 1 | 3 | 3,8 | 5,7 | 4,2 | 3,5 | 4,8 | 4,6 | 6,1 | 3,6 | 3,8 |
| 1% | 2 | 2,6 | 2,9 | 2,6 | 1,7 | 2,3 | 2,5 | 1,1 | 2,5 | 1,9 | 2 |
| 1% | 3 | 2,1 | 2,5 | 2,4 | 2,6 | 2,7 | 2,6 | 2,4 | 1,9 | 2,3 | 2,4 |
| 1% | 4 | 2,9 | 2,5 | 2,2 | 1,8 | 2,3 | 2,8 | 3,2 | 2,9 | 1,7 | 2,1 |
| 0,50% | 1 | 2,9 | 3,1 | 3,9 | 3,9 | 3,7 | 3,1 | 3,5 | 3,4 | 3 | 2,2 |
| 0,50% | 2 | 3,1 | 3,6 | 4 | 2,6 | 3,4 | 2,5 | 3,3 | 3,5 | 3,3 | 2,5 |
| 0,50% | 3 | 4,8 | 4,7 | 3,1 | 2,2 | 4,5 | 3,3 | 2,7 | 3,8 | 2,9 | 4,6 |
| 0,50% | 4 | 1,9 | 2,9 | 3,1 | 2,5 | 3,2 | 2,2 | 3,2 | 2,3 | 2,2 | 2,8 |
| 0,20% | 1 | 7,1 | 4 | 6,9 | 6,1 | 6,2 | 6,7 | 4,2 | 8,2 | 4,3 | 5,1 |
| 0,20% | 2 | 5,8 | 3,9 | 5,1 | 5,3 | 4,8 | 4,7 | 5,7 | 4,7 | 4,4 | 5 |
| 0,20% | 3 | 4,7 | 3,6 | 5,2 | 4,3 | 6,2 | 4 | 4,9 | 5,8 | 4,1 | 5,4 |
| 0,20% | 4 | 7,3 | 5 | 6,4 | 7,1 | 5,9 | 4,9 | 5,3 | 6,1 | 7,2 | 6,4 |
| 0,05% | 1 | 6,9 | 7,6 | 8,4 | 6,6 | 6,3 | 4,3 | 7,1 | 7 | 5,9 | 6 |
| 0,05% | 2 | 4,2 | 5,1 | 5,6 | 4,9 | 5,7 | 5,8 | 5,6 | 5,3 | 6,4 | 5,5 |
| 0,05% | 3 | 5,6 | 6 | 7,4 | 5,8 | 9,2 | 6,2 | 8,6 | 7,1 | 7 | 8,6 |
| 0,05% | 4 | 7,9 | 7,2 | 6,7 | 6,3 | 6,6 | 5,9 | 5,4 | 7,4 | 8 | 6,6 |

| *R. sativum* | | | | | | | | | | | |
|---|---|---|---|---|---|---|---|---|---|---|---|
| | | seed germination | | | | | | | | | |
| treatment | repetition | 1 | 2 | 3 | 4 | 5 | 6 | 7 | 8 | 9 | 10 |
| control | 1 | 21 | 23 | 23 | 25 | 25 | 25 | 25 | 25 | 25 | 25 |
| control | 2 | 22 | 24 | 24 | 24 | 24 | 24 | 24 | 24 | 24 | 24 |
| control | 3 | 22 | 22 | 22 | 24 | 25 | 25 | 25 | 25 | 25 | 25 |
| control | 4 | 24 | 25 | 25 | 25 | 25 | 25 | 25 | 25 | 25 | 25 |
| 2% | 1 | 25 | 25 | 25 | 25 | 25 | 25 | 25 | 25 | 25 | 25 |
| 2% | 2 | 23 | 23 | 24 | 24 | 25 | 25 | 25 | 25 | 25 | 25 |
| 2% | 3 | 24 | 25 | 25 | 25 | 25 | 25 | 25 | 25 | 25 | 25 |
| 2% | 4 | 22 | 25 | 25 | 25 | 25 | 25 | 25 | 25 | 25 | 25 |
| 1% | 1 | 6 | 24 | 25 | 25 | 25 | 25 | 25 | 25 | 25 | 25 |
| 1% | 2 | 16 | 24 | 24 | 25 | 25 | 25 | 25 | 25 | 25 | 25 |
| 1% | 3 | 13 | 25 | 25 | 25 | 25 | 25 | 25 | 25 | 25 | 25 |
| 1% | 4 | 13 | 25 | 25 | 25 | 25 | 25 | 25 | 25 | 25 | 25 |
| 0,50% | 1 | 21 | 24 | 24 | 24 | 25 | 25 | 25 | 25 | 25 | 25 |
| 0,50% | 2 | 22 | 24 | 24 | 24 | 25 | 25 | 25 | 25 | 25 | 25 |
| 0,50% | 3 | 21 | 24 | 24 | 25 | 25 | 25 | 25 | 25 | 25 | 25 |
| 0,50% | 4 | 25 | 25 | 25 | 25 | 25 | 25 | 25 | 25 | 25 | 25 |
| 0,20% | 1 | 23 | 24 | 24 | 24 | 25 | 25 | 25 | 25 | 25 | 25 |

| | | | | | | | | | | | |
|---|---|---|---|---|---|---|---|---|---|---|---|
| 0,20% | 2 | 23 | 24 | 24 | 24 | 24 | 25 | 25 | 25 | 25 | 25 |
| 0,20% | 3 | 22 | 24 | 25 | 25 | 25 | 25 | 25 | 25 | 25 | 25 |
| 0,20% | 4 | 23 | 25 | 25 | 25 | 25 | 25 | 25 | 25 | 25 | 25 |
| 0,05% | 1 | 22 | 23 | 24 | 24 | 24 | 24 | 24 | 24 | 24 | 24 |
| 0,05% | 2 | 25 | 25 | 25 | 25 | 25 | 25 | 25 | 25 | 25 | 25 |
| 0,05% | 3 | 20 | 22 | 23 | 23 | 23 | 24 | 24 | 24 | 24 | 24 |
| 0,05% | 4 | 22 | 24 | 24 | 24 | 24 | 24 | 24 | 25 | 25 | 25 |

| | | root lenght | | | | | | | | | |
|---|---|---|---|---|---|---|---|---|---|---|---|
| treatment | repetition | 1 | 2 | 3 | 4 | 5 | 6 | 7 | 8 | 9 | 10 |
| control | 1 | 15 | 16 | 12,5 | 14,7 | 15 | 13,5 | 17 | 10 | 15 | 13,3 |
| control | 2 | 12 | 18 | 12,3 | 16,5 | 15,6 | 12,2 | 11,5 | 15,5 | 11,5 | 16,5 |
| control | 3 | 13,5 | 13,5 | 11 | 8 | 17,5 | 12,3 | 19 | 12,5 | 20,21 | 17 |
| control | 4 | 17,5 | 17 | 13,5 | 12 | 11,5 | 11 | 16 | 10,5 | 14,5 | 12,5 |
| 2% | 1 | 8,2 | 7,5 | 4,5 | 3,5 | 4,5 | 4,2 | 4 | 7,5 | 4 | 3 |
| 2% | 2 | 7 | 8,5 | 4 | 6,5 | 6,5 | 4 | 6,5 | 5 | 5 | 5,7 |
| 2% | 3 | 5,7 | 4 | 7 | 4,2 | 2,2 | 5,3 | 6,9 | 5 | 3,8 | 5,5 |
| 2% | 4 | 4,2 | 6 | 4,5 | 5,5 | 4 | 6,5 | 3,3 | 7,7 | 4,5 | 3,5 |
| 1% | 1 | 8,2 | 4 | 4,7 | 4,9 | 8,4 | 5,2 | 11,5 | 8,4 | 9 | 5,6 |
| 1% | 2 | 7,4 | 8,8 | 9,1 | 8,4 | 7,3 | 11,9 | 10,7 | 9,4 | 8 | 9,2 |
| 1% | 3 | 16,4 | 10,6 | 8,5 | 8,6 | 11,1 | 12,5 | 10,2 | 13,5 | 6,5 | 8,7 |
| 1% | 4 | 6,5 | 7,3 | 10,3 | 12,6 | 10,1 | 5,5 | 9,4 | 9,4 | 8 | 9,2 |
| 0,50% | 1 | 8,8 | 7,7 | 7,3 | 8 | 10 | 6,9 | 6,5 | 5,5 | 10,3 | 9 |
| 0,50% | 2 | 9,9 | 8,5 | 7,5 | 7,2 | 8,7 | 9 | 7,2 | 5,8 | 6,5 | 6,1 |
| 0,50% | 3 | 7,5 | 7,8 | 8,7 | 7 | 6,7 | 7,7 | 8,2 | 8 | 8,9 | 7,2 |
| 0,50% | 4 | 7,8 | 6,2 | 7,2 | 9,2 | 9 | 8 | 6,5 | 9,5 | 7,5 | 8 |
| 0,20% | 1 | 9 | 8,5 | 8 | 7,7 | 8,4 | 6,4 | 6,5 | 7,5 | 8,5 | 10,2 |
| 0,20% | 2 | 10,7 | 8,5 | 11,5 | 8 | 8 | 6,5 | 6,5 | 7,5 | 7,2 | 9,5 |
| 0,20% | 3 | 9,4 | 12 | 10 | 9,2 | 9,7 | 9,2 | 8,6 | 6 | 9,5 | 8,1 |
| 0,20% | 4 | 7,2 | 8,2 | 6,5 | 6 | 7,5 | 9,3 | 8,5 | 9,5 | 8 | 9,5 |
| 0,05% | 1 | 10,2 | 17,8 | 16 | 12,2 | 8 | 8,5 | 21 | 10,3 | 8,5 | 9 |
| 0,05% | 2 | 12 | 11,7 | 11,3 | 6,5 | 7,5 | 8,5 | 12,5 | 14,2 | 14,5 | 13,5 |
| 0,05% | 3 | 15,5 | 17,7 | 13,2 | 15 | 12,5 | 13,5 | 11,5 | 6,3 | 8,5 | 7,5 |
| 0,05% | 4 | 17,2 | 14 | 12 | 16 | 10 | 10,2 | 14,5 | 12 | 8 | 11,5 |
